# A hospital-based matched case–control study to identify clinical outcome and risk factors associated with carbapenem-resistant *Klebsiella pneumoniae* infection

**DOI:** 10.1186/1471-2334-13-80

**Published:** 2013-02-11

**Authors:** Luci Correa, Marines Dalla Valle Martino, Itacy Siqueira, Jacyr Pasternak, Ana Cristina Gales, Claudia Vallone Silva, Thiago Zinsly Sampaio Camargo, Patricia Faria Scherer, Alexandre Rodrigues Marra

**Affiliations:** 1 Infection Control Unit, Hospital Israelita Albert Einstein, Av. Albert Einstein, 627/701 6º Andar Bloco D, São Paulo, ZIP 05652-000, Brazil; 2 Laboratory of Microbiology, Hospital Israelita Albert Einstein, Av. Albert Einstein, 627/701, São Paulo, ZIP 05651-901, Brazil; 3 Division of Infectious Diseases, Universidade Federal de São Paulo (UNIFESP/EPM), Rua Napoleão de Barros, 715, 7º andar, São Paulo, ZIP 04024-002, Brazil; 4 Intensive Care Unit, Hospital Israelita Albert Einstein, Av. Albert Einstein, 627/701, São Paulo, ZIP 05651-901, Brazil

**Keywords:** Klebsiella pneumoniae, Carbapenem-resistant *Klebsiella*, Healthcare associated infections, *Klebsiella* infections/microbiology, *Klebsiella* infections/mortality

## Abstract

**Background:**

Healthcare-associated infections caused by *Klebsiella pneumoniae* isolates are increasing and few effective antibiotics are currently available to treat patients. We observed decreased carbapenem susceptibility among *K. pneumoniae* isolated from patients at a tertiary private hospital that showed a phenotype compatible with carbapenemase production although this group of enzymes was not detected in any sample. The aim of this study was to describe the epidemiology and clinical outcomes associated with carbapenem-resistant *K. pneumoniae* and to determine the antimicrobial resistance mechanisms.

**Methods:**

Risk factors associated with carbapenem-resistant *K. pneumoniae* infections were investigated by a matched case–control study from January 2006 through August 2008. A cohort study was also performed to evaluate the association between carbapenem resistance and in-hospital mortality. Bacterial identification and antimicrobial susceptibility were determined by Vitek 2 and Etest. Carbapenemase activity was detected using spectrophotometric assays. Production of beta-lactamases and alterations in genes encoding *K. pneumoniae* outer membrane proteins, OmpK35 and OmpK36, were analyzed by PCR and DNA sequencing, as well as SDS-Page. Genetic relatedness of carbapenem resistant isolates was evaluated by Pulsed Field Gel Electrophoresis.

**Results:**

Sixty patients were included (20 cases and 40 controls) in the study. Mortality was higher for patients with carbapenem-resistant *K. pneumoniae* infections compared with those with carbapenem-susceptible *K. pneumoniae* (50.0% vs 25.7%). The length of central venous catheter use was independently associated with carbapenem resistance in the multivariable analysis. All strains, except one, carried *bla*_CTX-M-2,_ an extended-spectrum betalactamase gene. In addition, a single isolate also possessed *bla*_GES-1._ Genes encoding plasmid-mediated AmpC beta-lactamases or carbapenemases (KPC, metallo-betalactamases or OXA-carbapenemases) were not detected.

**Conclusions:**

The *K. pneumoniae* multidrug-resistant organisms were associated with significant mortality. The mechanisms associated with decreased *K. pneumoniae* carbapenem susceptibility were likely due to the presence of cephalosporinases coupled with porin alterations, which resulted from the presence of the insertion sequences in the outer membrane encoding genes.

## Background

Healthcare-associated infections, such as meningitis, pneumonia, and wound, surgical site and bloodstream infections caused by resistant gram-negative organisms such as *Klebsiella pneumoniae* are increasing [1]. This is a major public health threat and although antibiotics such as carbapenem can be used to treat ESBL *Klebsiella*, some strains of *Klebsiella* have developed resistance to carbapenem and polymyxin B is the option for this XDR *Klebsiella*[2,3]. In 2003, the National Nosocomial Infection Surveillance System reported a 47% increase over 5 years in third-generation cephalosporin resistance in *K. pneumoniae* isolates recovered from patients in intensive care units (ICUs) [4]. Extended-spectrum β-lactamase (ESBL) enzymes are produced by bacteria that render them resistant to antibiotic treatment. Currently, there is a worldwide spread of ESBL-producing *K. pneumoniae* isolates in hospitals [5-8]. In response to this, carbapenem use has increased, resulting in increased bacterial carbapenem resistance [9]. During 2006/2007 in the US, high percentages of carbapenem-resistant *K. pneumoniae* isolates (4 to 11% of pathogenic isolates, with higher resistance found in isolates of primary bloodstream infections) were identified in ICUs by the National Healthcare Safety Network [10].

Carbapenem-resistant *Enterobacteriaceae* (CRE) pose a particular challenge as they cause numerous diseases, are hard to treat and have the potential to spread within healthcare facilities. Infections with these organisms are associated with high rates of morbidity and mortality [11-17]. Bacterial resistance to carbapenems may involve several combined mechanisms, such as the hyperproduction of AmpC β lactamases (cephalosporinases) and/or production of ESBLs and/or specific carbapenem hydrolyzing enzymes (carbapenemases) associated with alterations in the bacterial outer membrane proteins and hyperexpression of efflux systems [9]. The production of carbapenemase enzymes (KPC enzymes) is the most important mechanism of resistance to carbapenems in *K. pneumoniae*. The *bla*_KPC_ gene is mostly plasmid-encoded and can be transferred to different *K. pneumoniae* clones and even to different bacterial genera [17].

Currently, *K. pneumoniae* is the most frequent species of CRE found in the United States and has recently been detected in Brazilian hospitals [18-20]. The outbreak and endemic dissemination of KPC-producing *K. pneumoniae* in hospitals is related to cross transmission with the predominance of few clones [12,21]. Contact precautions and active surveillance are common measures employed for controlling the spread of these microorganisms in hospitals [22].

We observed a decrease in carbapenem susceptibility among *K. pneumoniae* in the Hospital Israelita Albert Einstein, São Paulo, Brazil. Interestingly, carbapenemase production was not detected in these strains and thus we investigated the epidemiology and clinical outcomes associated with these pathogens and determined their mechanism of resistance to beta-lactam antibiotics.

## Methods

### Patients and data collection

This study was conducted at the Hospital Israelita Albert Einstein (HIAE), a 620-bed private tertiary hospital in São Paulo, Brazil. Approximately 36,000 patients are admitted each year and most ward patients are hospitalized in private rooms.

All patients presenting *K. pneumoniae* healthcare-associated infections (HAIs) during the period from January 2006 to August 2008 were identified through the HIAE infection control database. Healthcare-associated infections were defined as those acquired after 48 hours of hospital admission. Active surveillance of all HAIs was performed by trained infection control nurses using Centers for Disease Control and Prevention (CDC) definitions [23] in the following units: bone marrow transplantation unit, step-down units, adult intensive care, pediatric and neonatal intensive care units. All episodes of catheter-associated bloodstream infections and surgical site infections were actively surveyed in all wards. When a patient had multiple episodes of *K. pneumoniae* infection, only the first occurrence was included in the study. A matched case–control study was performed to identify risk factors associated with carbapenem-resistant *K. pneumoniae*. Patients presenting carbapenem-resistant *K. pneumoniae* infections were considered as belonging to the case group or classified under the case group and compared to the control group (patients infected by carbapenem-susceptible *K. pneumoniae*). Matching was performed at a ratio of 1:2 according to infection date, anatomic site of infection, and the unit where infection was acquired. The association between carbapenem resistance and in-hospital mortality was evaluated by a cohort study and included all patients (case and control subjects). Both study strategies were approved by the Research Ethics Committee of the Hospital Israelita Albert Einstein.

*K. pneumoniae* isolates with imipenem and/or meropenem minimum inhibitory concentrations (MICs) ≥ 2 μg/ml as assessed by the Vitek 2 automated system (BioMérieux, St Louis, MO) were screened as possible carbapenemase producers. The reduced susceptibility or resistance to carbapenems was confirmed by Etest (BioMérieux, Marcy-l'Etoile, France). The interpretative criteria for antimicrobial susceptibility testing were according to the Clinical Laboratory Standards Institute (CLSI) M100-S19 document [24].

Clinical data were obtained from medical records. Variables analyzed as risk factors included sex, age, level of comorbidity [25], underlying disease severity [26], and treatments and procedures performed prior to the infection (such as steroid administration, neutropenia, dialysis, solid-organ or hematopoietic stem cell transplantation, surgery, use of invasive devices, and exposure to antibiotics). Length of hospital stay and previous ICU hospitalization before infection acquisition were also considered. Illness severity was assessed according to APACHE II and SOFA scores for patients hospitalized in ICUs [27,28].

The Charlson score was divided into ≥ 3 (higher degree of underlying comorbidity), and ≤ 2 (lower degree). Antimicrobial usage was considered only when it occurred within 15 days prior to the infection diagnosis and its administration lasted for at least 48 hours [29]. Data regarding the antibiotics administered after isolation of *K. pneumoniae*, the time of administration, and whether the treatment was appropriate were collected. Adequate empiric antimicrobial treatment was defined as therapy administered within 24 hours after cultures samples were obtained that included the administration of an antimicrobial agent to which the *K. pneumoniae* isolate was resistant. Antimicrobial agents were considered adequate if the organism was susceptible, except when cephalosporins were used for the treatment of ESBL infections [30,31]. Septic shock was defined as sepsis associated with hypotension unresponsive to intravenous fluid challenge or requiring a vasopressor agent [32].

### Microbiological studies

Among the 20 *K. pneumoniae* isolates responsible for causing infection, 17 strains from 16 patients were viable after being stored at −80°C. For comparison purposes, a *K. pneumoniae* isolate susceptible to carbapenems was also included in the microbiological analysis because it was previously isolated from a patient who was subsequently infected by *K. pneumoniae* with reduced susceptibility to carbapenems .

Imipenem and meropenem MICs were confirmed by CLSI broth microdilution (TREK Diagnostic Inc. Westlake, OH). Quality control was performed by testing *Escherichia coli* ATCC 25922 and *Pseudomonas aeruginosa* ATCC 27853.

Hydrolysis of imipenem was assessed by UV spectrophotometric assays as previously described [33]. Briefly, a full 10 μl loop of the test organism was inoculated into 500 μl of phosphate buffer 100 mM (pH 7.0) and then disrupted by sonication. Whole protein extracts were obtained after centrifugation. Hydrolytic activity of 20 μl of the crude extract was determined against 100 μM imipenem in 100 mM phosphate buffer (pH 7.0), and measurements were carried out at a 297 nm wavelength.

Specific primers under standard PCR conditions were used to detect ESBL- and carbapenemase encoding genes, namely, *bla*_TEM_*, bla*_SHV_, *bla*_CTX-M_*, bla*_GES_, *bla*_KPC_, *bla*_IMP_*, bla*_VIM_, *bla*_SPM_*, bla*_GIM_, *bla*_SIM_*, bla*_OXA-2_, *bla*_OXA-10_*, bla*_OXA-23_*, bla*_OXA-24_, *bla*_OXA-48_, *bla*_OXA-51_, *bla*_OXA-58_[34-37]. Amplified DNA fragments were purified with the Qiaquick PCR purification kit (Quiagen, Courtaboeuf, France) and sequenced on both strands with an automated ABI 337 sequencer (Applied Biosystems, Foster City, USA). The nucleotide and deduced protein sequences were analyzed with software available over the internet at the National Center for Biotechnology Information website. Alteration in the *ompK35* and *ompK36* genes that encode the *K. pneumoniae* major outer membrane porins were investigated by PCR and DNA sequencing [38]. Sixteen carbapenem-resistant *K. pneumoniae* isolates were sequenced for the *ompk35* and *ompk36* genes, as well as evaluated the OMP profiles by sodium dodecyl sulfate-polyacrylamide gel electrophoresis (SDS-PAGE) [39]. Pulsed field gel electrophoresis (PFGE) was performed using SpeI. The results were interpreted following the criteria of Tenover *et al*. [40].

### Statistical analysis

For continuous variables, mean values were compared using two sample *t*-tests for independent samples. The Mann–Whitney *U*-test was performed for non-normally-distributed continuous variables. Differences in proportions were compared using a Chi-square test or Fisher’s exact test when appropriate. Mean values are reported ± 1 SD. All tests of significance are two-tailed. When collinearity was identified between two variables, the one with the greatest clinical relevance associated with the event was included in the multivariate analysis. Odds ratios (OR) were calculated for independent variables associated with in-hospital mortality among patients with *K. pneumoniae* infections and for independent variables associated with carbapenem-resistant *K. pneumoniae*. The association of independent variables was expressed as OR with 95% confidence intervals. Alpha was set at 0.05. All statistical analyses were performed using the Statistical Package for the Social Sciences software (SPSS 17.0, Chicago, IL, USA).

## Results

A total of 236 episodes of *K. pneumoniae* healthcare-associated infections were diagnosed in 175 patients during the study period. Twenty patients had carbapenem-resistant *K. pneumoniae* infections (8.5%), an overall rate of 0.7 episodes of carbapenem-resistant *K. pneumoniae* nosocomial infections per 10,000 patient-days. The carbapenem-resistant *K. pneumoniae* infections were: urinary tract infections (9), central venous catheter-associated bloodstream infections (5), surgical site infections (4) and skin and soft tissue infections (2).

There were no significant differences (p > 0.05) among cases and controls, with regard to baseline demographic and clinical characteristics. Among the 20 patients with imipenem and/or meropenem-resistant *K. pneumoniae* infections, 10 (50.0%) died during hospitalization, while 11 (27.5%) of the 40 patients with carbapenem-susceptible *K. pneumoniae* infections (p = 0.085) died.

By univariable analysis (Table 1) carbapenem-resistant *K. pneumoniae* infection was associated with previous ICU stay, central venous catheter catheterization, a longer central venous catheter use, and exposure to antimicrobials. By multivariable analysis only the length of central venous catheter use was independently associated with carbapenem resistance (OR 1.08 [95% CI, 1.01-1.16]).

**Table 1:** **Summary of risk factors associated with carbapenem-resistant *K. pneumoniae *infection**

**Risk factor**	**Case patients (n = 20)**	**Control patients (n = 40)**	**Univariable analysis**		**Multivariable analysis**	
			**OR (95% CI)**	**p**	**OR (95% CI)**	**p**
Male sex	13 (65.0)	21 (52.5)	0.59 (0.19-1.81)	0.36		
Mean Age (years)	59.6	64.9	5.35 (−6.89-17.59)	0.38		
**McCabe score**						
Rapidly fatal	4 (20.0)	11 (27.5)	1.52 (0.42-5.55)	0.75		
Potentially fatal/Non-fatal	16 (80.0)	29 (72.5)				
Charlson score ≥ 3	11 (55.0)	12 (30.0)	2.85 (0.94-8.66)	0.06		
Transplant receipt	7 (35.0)	6 (15.0)	0.33 (0.09-1.16)	0.10		
Prior corticosteroid use	16 (80.0)	22 (55.0)	0.31 (0.09-1.08)	0.06		
Prior surgery	14 (70.0)	22 (55.0)	0.53 (0.17-1.64)	0.27		
Dialysis	6 (30.0)	6 (15.0)	0.42 (0.12-1.49)	0.17		
ICU stay*	20 (100)	32 (80.0)	0.62 (0.49-0.76)	0.03	-	-
Mean APACHE II score at admission	22.1	16.4	1.10 (1.02-1.19)	0.02	1.10 (0.97-1.25)	0.13
Mean SOFA score at admission	7.35	5.63	1.14 (0.98-1.32)	0.09		
Mean length of stay before infection (days)	45.5	27		0.11		
**Device use**						
CVC	17 (85.0)	23 (57.5)	4.18 (1.10-16.7)	0.04	0.05 (0.01-2.20)	0.12
Mechanical ventilation	4 (20.0)	5 (12.5)	0.57 (0.13-2.42)	0.46		
Urinary catheter	12 (60.0)	29 (72.5)	1.56 (0.51-4.77)	0.44		
**Mean device use (days)**						
CVC						
Urinary catheter	23.5	12.2		0.02	1.07 (0.99-1.16)	0.07
Mechanical ventilation	13.0	17.1		0.56		
	21.0	22.0		0.98		
Prior antimicrobial use**	20 (100)	31 (77.5)	1.67 (1.32-2.05)	0.02	-	1.0

Univariable analysis shows carbapenem-resistant *K. pneumoniae* infection was associated with previous ICU stay, central venous catheter catheterization, a longer central venous catheter use, and exposure to antimicrobials. Multivariable analysis demonstrated that only the length of central venous catheter was independently associated with carbapenem resistance.

*The variable ICU stay was constant for all selected cases. Since a constant was requested in the model, it was removed from the analysis.

**The prior use of antimicrobial agents was considered independently of the antimicrobial class used, for the multivariate analysis.

Abbreviations used: CVC, central venous catheter.

Figures in parentheses represent percentage values unless otherwise stated.

Risk factors associated with in-hospital mortality among patients with *K. pneumoniae* infection measured by univariable analysis included receiving dialysis, elevated APACHE scores, and vasopressor drug administration and were predictors of death (Table 2). The prescription of an adequate initial antimicrobial regimen according to susceptibility testing results was not associated with patient survival. The appropriate definitive antimicrobial therapy was delayed in 3 days (median) in both groups, case and control patients.

**Table 2:** **Summary of univariable analysis of risk factors associated with mortality among patients with *K. pneumoniae *infections**

**Risk factor**	**Survivors (n = 39)**	**Non-survivors (n = 21)**	**OR (95% CI)**	**p**
Male sex	20 (51.3)	14 (66.7)	0.53 (0.16-1.59)	0.25
**McCabe score**				
Rapidly fatal	10 (25.6)	5 (23.8)	1.1 (0.32-3.79)	0.15
Potentially fatal/Non fatal	29 (74.4)	16 (76.2)		
Charlson score ≥ 3	13 (33.3)	10 (47.6)	1.82 (0.62-5.38)	0.28
Transplant receipt	7 (17.9)	6 (28.6)	0.55 (0.16-1.91)	0.35
Prior corticosteroid use	24 (61.5)	14 (66.7)	0.80 (0.26-2.44)	0.69
Prior surgery	27 (69.2)	10 (47.6)	2.48 (0.83-7.39)	0.10
Dialysis	4 (10.3)	8 (38.1)	0.19 (0.05-0.72)	0.01
ICU stay	32 (82.1)	20 (95.2)	0.23 (0.03-1.99)	0.15
APACHE II score, mean, on admission	16.2	22.4	1.39 (−10.65-1.65)	0.009
SOFA score, mean, on admission	5.6	7.7	3.59 (−4.54 -0.27)	0.08
Vasopressor drug use	1 (2.6)	6 (28.6)	15.2 (1.68-137.15)	0.006
Mechanical ventilation	5 (12.8)	7 (33.3)	3.40 (0.92-12.55)	0.09
Appropriate antibiotic therapy	27 (69.2)	11 (52.4)	2.04 (0.68-6.11)	0.19
Carbapenem resistance	10 (25.6)	10 (47.6)	2.64 (0.86-8.07)	0.085

Univariable analysis demonstrated that risk factors associated with in-hospital mortality among patients with *K. pneumoniae* infection included receiving dialysis, elevated APACHE scores, and vasopressor drug administration. Figures in parentheses represent percentage values unless otherwise stated.

The results of the GES, CTX-M enzyme production, and the amplicon resulted of PCR amplification of the *ompk*35 and *ompk*36 are shown in Table 3. Among the 17 *K. pneumoniae* isolates evaluated, no carbapenem hydrolysis was detected by spectrophotometric assay or the presence of genes encoding plasmid-mediated AmpC beta-lactamase or carbapenemases. Only one strain revealed the presence of the *bla*_GES-1_ gene and all strains except one showed *bla*_CTX-M-2_, genes responsible for codifying ESBL. PCR analysis of OMP-encoding genes showed altered amplicons of at least one OMP-encoding gene, including a lack of amplification (8 isolates, 47.1%) or enhanced amplicon size (9 isolates, 53.0%). Through the DNA sequencing of some of these amplicons (data not shown), we observed that acquisition of insertion sequences (IS) were responsible for the unexpected, higher molecular size of the *ompK35* or ompk36 amplification. Mutations on OmpK35- and/or OmpK36-encoding genes was observed in all isolates presenting only the constitutively OmpA in the SDS gels, such as a premature stop codon or a insertion sequence disrupting the porin gene. Seven different PFGE patterns were observed with a predominance of one subtype. This predominant subtype was observed in seven patients. Among these patients only two have been in the same intensive care unit for six days, although this period of companionship was about two months before the onset of the infection.

**Table 3:** Resistant mechanisms detected in different strains

**Strain**	**GES**	**CTX-M 2**	**ompK35**	**ompk36**
1	NEG	**POS**	**>2072**	**>2072**
2	NEG	**POS**	**>2072**	NA
3	NEG	**POS**	**>2072**	**>2072**
4	NEG	**POS**	**>2072**	NA
5	NEG	**POS**	1000	**>2072**
6	NEG	**POS**	**>2072**	NA
7	NEG	**POS**	**>2072**	1000
8	NEG	**POS**	1000	1000
9	**POS**	**POS**	1000	**>2072**
10	NEG	**POS**	1000	NA
11	NEG	**POS**	1000	NA
12*	NEG	NEG	1000	NA
13*	NEG	**POS**	1000	1000
14	NEG	NEG	1000	1000
15	NEG	**POS**	NA	1000
16	NEG	**POS**	**>2072**	1000
17	NEG	**POS**	1000	NA

* The isolates 12 (susceptible strain) and 13 (resistant strain) are from the same patient.

Analysis of the clinical isolate from a patient who initially harbored a *K. pneumoniae*-susceptible strain and then subsequently presented with a *K. pneumoniae*-resistant strain, demonstrated that although this strain belonged to the same PFGE subtype, it had no amplification of *ompk36* gene. It suggests that the patient remained infected by the same *K. pneumoniae* clone that had become resistant to carbapenems probably due to the acquisition of an ESBL encoding gene and loss of OmpK36.

## Discussion

Carbapenem resistance among the *Enterobacteriaceae* is an emerging phenomenon of vast clinical and public health importance. Controlling the spread of KPC enzymes is difficult once the gene encoding this enzyme reside on transmissible plasmids [23]. Current automated susceptibility testing methods have failed to reliably detect carbapenem resistance among *K. pneumoniae* isolates [38]. In this study, we obtained MICs for *K. pneumoniae* isolates by broth microdilution, thus avoiding the misclassification of some case patients as potential control subjects. During the study period, all *K. pneumoniae* isolates with MIC ≥ 2 μg/ml detected by the Vitek system were submitted for other susceptibility tests and screened for KPC production. *K. pneumoniae* isolates harboring KPC enzymes had MICs for carbapenem in a range that allowed *K. pneumoniae* to remain susceptible to carbapenem, and could therefore go unrecognized. Since 2010 CLSI have changed breakpoints and *Enterobacteriaceae* isolates with MICs for imipenem and/or meropenem ≥ 2 μg/ml have been categorized as intermediate or resistant (CLSI, M100-S20-U). Therefore, some strains included in our study would be classified as imipenem and/or meropenem resistant if the most recent CLSI breakpoints are applied.

The presence of a premature stop codon in the porin gene could explain why some *K. pneumoniae* isolates included in the present study presenting an *ompK35* or *ompK36* amplicon size of 1000 bp are resistant to carbapenens.

During the 32 months of the study period, 20 patients were diagnosed with healthcare-associated infections caused by carbapenem-resistant *K. pneumoniae* strains in our hospital. This represented 8.5% of the total episodes of *K. pneumoniae* healthcare-associated infections. Analysis of data on infectious disease outcomes of patients revealed that carbapenem-resistant *K. pneumoniae* patients had a higher mortality compared with patients infected with carbapenem-susceptible *K. pneumoniae* (50.0% and 27.5%, respectively), although it was not statistically significant (p=0.085). Similar harmful effects on patient outcomes have been observed in previous studies where carbapenem-resistant *K. pneumoniae* associated mortality was between 30 and 50% [11,12,15,16,21,41,42]. These studies examined the epidemiology of KPC producers during *K. pneumoniae* related-infections. Of interest, although patients included in our study were infected by carbapenem-resistant *K. pneumoniae* strains that did not produce KPC carbapenemase, they had similar outcomes in terms of mortality.

Evaluation of the factors that predict carbapenem resistance by univariable analysis, demonstrated that prior ICU stay, central venous catheterization, longer use of a central venous catheter, and exposure to antimicrobials were associated with carbapenem-resistant *K. pneumoniae* infection. In the multivariable analysis only the length of central venous catheter use was independently associated with *K. pneumoniae* carbapenem resistance. Previous studies reported similar risk factors for carbapenem-resistant *K. pneumoniae* infection and demonstrated associations with length of hospital stay, ICU admission, use of central venous catheter, recent solid-organ or stem-cell transplantation, receipt of mechanical ventilation, and exposure to broad-spectrum antibiotics [11,12,15]. Overestimation of the importance of antibiotic exposure as a risk factor is a common selection bias in case–control studies in which control subjects have susceptible isolates. Surprisingly, in this study carbapenem use was not an independent predictor for carbapenem resistance. This unexpected finding may be related to the small sample size of this study.

To further explore the risk of mortality in *K.* pneumoniae-infected patients (both case studies and controls), we evaluated the impact of patient characteristics and treatment interventions. Unexpectedly, the initial treatment of patients with antibiotics for clinical isolates that were *in vitro* susceptible to treatment was not associated with patient survival.

Therefore, poor patient outcomes cannot be fully explained by a delay in providing the appropriate therapy. Previous studies have suggested that removal of the focus of infection, such as a catheter, debridement, or drainage, is an effective way of improving survival among patients with carbapenem-resistant *K. pneumoniae* infections [11]. However, this adjunctive therapy was not evaluated in our study.

Besides observing clinical characteristics, we also performed molecular analysis of isolated *K. pneumoniae* strains to analyze the mechanisms of antimicrobial resistance, and to rule out the possibility of an outbreak during the study period. Although carbapenemase encoding genes including *bla*_KPC_ were not identified in any of the *K. pneumoniae* isolates studied, *bla*_CTX-M-2_ and *bla*_GES-1,_ ESBL encoding genes were detected in our collection. In addition, we observed changes in OMP-encoding genes amplicon size by PCR in 9 isolates suggesting the likelihood of altered porin functions. The amplicon size expected for *ompK35* or ompk36 amplification is around 1000 bp. Through the DNA sequencing of some of these amplicons, we observed that acquisition of insertion sequences were responsible for the unexpected, higher molecular size of the *ompK35* or ompk36 amplification. Therefore, we conclude that impermeability of outer membrane proteins contributed considerably to carbapenem decrease susceptibility in those *K. pneumoniae* isolates, especially when these isolates were ESBL producers (especially CTX-M-2- producing *K. pneumoniae*).

Most cases did not cluster in time and space. Molecular epidemiology revealed that the isolates belonged to seven distinct clones, although one subtype was predominant. However, most cases could not be linked to a specific patient-to-patient transmission event or to a common source.

Our results show that cephalosporinase production associated with porin modifications likely contributed to carbapenem resistance. This study focused on bacterial infection not colonization, and this allowed for a more accurate analysis of prognosis and mortality, as we only included patients with ongoing infections.

There were a number of limitations in this study. First, we had a low number of episodes of carbapenem-resistant *K. pneumoniae* infections in our hospital, suggesting the number of samples analyzed was small. Moreover this sample size may be underpowered to detect small significant differences. Second, we were not able to include prior colonization with carbapenem-resistant *K. pneumoniae* in our risk factor analysis for invasive infection, because the colonization status of each patient was unknown. At that time point we did not perform active surveillance for carbapenem-resistant *Enterobacteriaceae* (by rectal or peri-rectal swabs). Third, the case–control design for analyzing the risk factors for antimicrobial resistance has some limitations. The use of patients infected with carbapenem-susceptible *K. pneumoniae* as control subjects may be falsely inflated prior antimicrobial exposure (which was not observed in our study). The ability to match control-patients on important variables, such as time at risk and location, is problematic in case–control studies. Finally, because this study was performed at a single medical center, these results may not extrapolate to other hospitals.

## Conclusions

In conclusion, the analysis of antimicrobial resistance and the molecular characterization of *K. pneumoniae* were useful for understanding preventive measures that should be implemented in the hospital.

## Competing interests

The authors declare that they have no competing interests.

## Authors’ contributions

LC was the study’s main investigator, participated in its design and drafted the manuscript. MDVM and IS performed the phenotype analysis. ACG carried out the molecular genetic studies, performed the PFGE analysis and helped to review the manuscript. CVS and TZSC were responsible for reviewing the infection control database. PFS reviewed the medical records, collected the epidemiological data and participated in the statistical analysis. JP participated in the design and provided expert oversight. ARM conceived of the study, performed the statistical analysis and helped to draft the manuscript. All authors read and approved the final manuscript.

## Pre-publication history

The pre-publication history for this paper can be accessed here:

http://www.biomedcentral.com/1471-2334/13/80/prepub
